# Pneumococcal meningitis: analysis of the cases admitted in the last year in the intensive care unit of the National Institute for Infectious Diseases “Prof. Dr. Matei Balş”, Bucharest

**DOI:** 10.1186/1471-2334-13-S1-P99

**Published:** 2013-12-16

**Authors:** Andrei Rogoz, Cleo Roşculeț, Ana-Maria Petrescu, Cătălin Apostolescu, Monica Ivan, Marius Radu, Aida Răşcanu, Alina Frangulea, Monica Ungureanu, Mihaela Tudor, Bogdan Pavel, Doina Iovănescu

**Affiliations:** 1 National Institute for Infectious Diseases “Prof. Dr. Matei Balş”, Bucharest, Romania

## Background

Invasive pneumococcal infections are still associated with significant morbidity and mortality. The objective of this work is to emphasize the correct therapeutic approach in pneumococcal meningoencephalitis.

## Methods

We retrospectively analyzed a series of patients admitted in the National Institute for Infectious Diseases “Prof. Dr. Matei Balş” Intensive Care Unit (ICU) between September 2012 and September 2013, regarding the underlying diseases, clinical and biological features, the treatment, the complications and the outcome. We reviewed the antimicrobial guidelines regarding the pneumococcal invasive infections.

## Results

In one year interval there were 7 cases of severe pneumococcal meningoencephalitis admitted in Matei Balş ICU, Adults Department. All the pneumococcal strains identified were penicillin sensitive. Despite broad antibiotic coverage, after admission, the evolution was severe and the mortality rate was around 70%. Delayed hospital presentation and/or diagnosis explained the majority of the therapeutic failures.

## Coclusion

Proper treatment of the entities that can lead to pneumococcal meningoencephalitis (as ENT infections), prompt recognition of the first symptoms and rapid accurate treatment offer the best chance of a good outcome.

